# The rising incidence of severe sepsis based on administrative data — real change or coding-driven bias?

**DOI:** 10.1186/s13054-015-1113-4

**Published:** 2015-11-12

**Authors:** Lavi Oud

**Affiliations:** Division of Pulmonary and Critical Care Medicine, Department of Internal Medicine, Texas Tech University Health Sciences Center at the Permian Basin, Odessa, TX 79763 USA

## Letter

Rhee and colleagues conclude in their recent study [1] that a substantial fraction of the administrative data-based rise in the incidence of severe sepsis is due to a decreasing threshold of documentation/coding of several types of organ dysfunction (OD). The conclusion was based mostly on diverging rates of rise in code-based OD versus that based on conservative clinical criteria, being faster among the former, coupled with a slower drop in hospital mortality among the latter.

However, these findings do not preclude an actual rise in the incidence of severe sepsis as a key driver of the observed trends, with reduced peak severity of sepsis-associated OD over time, possibly related to increasingly timely and effective resuscitative interventions. Indeed, the investigators reported a clinical criteria-based rising incidence of shock (+29 %) versus concomitant drop (−27 %) of acute renal failure. The latter finding may indicate that evolving renal failure has become increasingly less severe than the study’s definition threshold, despite the marked rise in shock.

Finally, the slower decrease in hospital mortality among clinical criteria-based OD may alternatively represent a higher likelihood of in-hospital death among patients with more severe OD, while those with relatively less severe OD may be more likely to be discharged to a long-term care facility or hospice, shifting in part their location of death. The later proposition is supported by prior reports [2] and a recent study by Kumar et al. [3] showing that the absolute drop in severe sepsis-associated hospital mortality (12.3 %) was matched by a similar rise (11.6 %) in discharge to a long-term care facility or hospice.

## Abbreviation

OD: Organ dysfunction
